# Clinical meaning of age-related expression of fecal cytokeratin 19 in colorectal malignancy

**DOI:** 10.1186/1471-2407-9-376

**Published:** 2009-10-22

**Authors:** Chun-Chao Chang, Shung-Haur Yang, Chih-Cheng Chien, Shu-Hung Chen, Shiann Pan, Chia-Long Lee, Chih-Ming Lin, Hsiao-Lun Sun, Chi-Cheng Huang, Yih-Yiing Wu, Ruey-Neng Yang, Chi-Jung Huang

**Affiliations:** 1 Digestive Disease Research Center, Taipei Medical University and Division of Gastroenterology, Department of Internal Medicine, Taipei Medical University Hospital, Taipei 11031, Taiwan, Republic of China; 2 Department of Surgery, Taipei-Veterans General Hospital and School of Medicine, National Yang Ming University, Taipei 11217, Taiwan, Republic of China; 3 Department of Anesthesiology, Sijhih Cathay General Hospital, Sijhih City, Taipei 22174, Taiwan, Republic of China; 4 Department of Surgery, Hsinchu Cathay General Hospital, Hsinchu 30060, Taiwan, Republic of China; 5 Department of Internal Medicine, Cathay General Hospital, Taipei 10630, Taiwan, Republic of China; 6 Department of Surgery, Cathay General Hospital, Taipei 10630, Taiwan, Republic of China; 7 Department of Pathology, Cathay General Hospital, Taipei 10630, Taiwan, Republic of China; 8 School of Medicine, Fu Jen Catholic University, Taipei 24205, Taiwan, Republic of China; 9 Department of Biochemistry, National Defense Medical Center, Taipei 11490, Taiwan, Republic of China; 10 Department of Internal Medicine, Sijhih Cathay General Hospital, Sijhih City, Taipei 22174, Taiwan, Republic of China; 11 Department of Surgery, Sijhih Cathay General Hospital, Sijhih City, Taipei 22174, Taiwan, Republic of China; 12 Department of Medical Research, Cathay General Hospital, Taipei 10630, Taiwan, Republic of China; 13 Institute of Biomedical Electronics and Bioinformatics, National Taiwan University, Taipei 10617, Taiwan, Republic of China

## Abstract

**Background:**

Colorectal cancer (CRC) is one of the leading causes of malignant death worldwide. Because young age of onset is often considered a poor prognostic factor for CRC, it is important to identify the poor outcomes of CRC in a younger population and to consider an aggressive approach by implementing early treatment. Our aim was to specifically quantify the fecal cytokeratin 19 (CK19) transcript from CRC patients and investigate its correlation with clinical stage, tumor malignancy, and age.

**Methods:**

The quantitation of fecal CK19 transcript was determined by a quantitative real-time reverse transcription polymerase chain in 129 CRC patients (45 younger than 60 years at diagnosis) and 85 healthy controls. The levels of CK19 protein were examined both in colonic cell lines and tissues.

**Results:**

The analysis of 45 younger CRC patients (age ≤ 60 years) revealed that patients at the M1 stage had significantly higher expression levels of fecal CK19 mRNA when compared with healthy controls (*p *< 0.001) and patients at the M0 stage (*p *= 0.004). Additionally, the degree of consistency between the mean level of fecal CK19 mRNA and the distant metastatic rate in each age interval was up to 89% (*p *= 0.042).

**Conclusion:**

These results indicate that high levels of fecal CK19 mRNA represent a potential marker for colorectal malignancy and for aggressive treatment of younger CRC patients.

## Background

Colorectal cancer (CRC), which is a predominant gastrointestinal malignancy, is one of the most commonly diagnosed tumors in both men and women, and is becoming one of the major medical causes of economic burden worldwide [1]. On average, the starting age of CRC incidence begins at 40 years of age and rises sharply at the age of 50-55 years [2]. Moreover, CRC is also the second most common cause of cancer-related deaths among men over 40 years of age [3].

Several clinicopathological features of CRC have been studied to identify markers that could predict CRC outcomes [4]. Numerous studies have shown that metastasis through the blood or lymphatic vessels is a major complication of cancer, and affects the prognosis of patients with primary carcinomas [5], therefore, methods developed to detect disseminated tumor cells in the peripheral blood and lymph nodes of patients have been evaluated. Many genetic changes were found in metastatic tumors, and some of them could be molecular markers for disseminated tumor cells [6]. CRC development and progression were shown to be complex processes that are associated with multiple genetic alterations [7]. One of these mutant molecules, cytokeratin 19 (CK19), is differentially expressed in the peripheral blood [8,9] and lymph nodes [10] of patients with breast cancer, or in epithelial cells of CRC patients with advanced Dukes' stage [11]. In addition, serum levels of the CK19 protein fragment CYFRA 21-1 were also evaluated in many cancers, and could represent a useful circulating tumor marker [12-14].

Because young age of onset is often considered a poor prognostic factor for CRC [15,16], it is important to identify the poor outcomes of CRC in a younger population and to consider an aggressive approach by implementing early treatment [17], therefore, a potential marker that allows the evaluation of colorectal malignancy in young patients is necessary. In the search for CRC biomarkers, many studies have suggested that a molecular test using fecal material may allow the elucidation of the molecular pathogenesis of CRC [18,19]. We previously reported that the upregulation of CK19 in feces predicted the presence of metastasis [20].

In the present study, we used quantitative real-time reverse transcription polymerase chain reaction (qRT-PCR) [21] to specifically quantify the CK19 transcript, which is considered to be relatively specific for epithelial cells in the feces of CRC patients. We also investigated the correlation between fecal CK19 mRNA transcript levels and clinical stage, tumor malignancy, and age.

## Methods

### Patients

One hundred twenty-nine CRC patients from Taipei Veterans General Hospital and Cathay General Hospital provided informed consent to participate in this study, which complied with the guidelines approved by the institutional review boards. The mean age of the patients was 65 years (age range, 32-90 years) and the cohort included 79 males and 50 females. Their initial tumor stage and other clinical characteristics were listed in Table 1. Abdominal computed tomography (CT) was routinely performed to monitor for the presence of metastasis; however, chest CT was only performed in cases with suspected lung lesions.

**Table 1:** Characteristics of CRC patients and healthy controls

Factor	Category	CRC patients	Healthy controls
Age (years)	*n*	129	85
	Mean (range)	65 (32-90)	57 (32-91)
Gender	*n*	129	85
	Male (%)	79 (61.2)	42 (49.4)
	Female (%)	50 (38.8)	43 (50.6)
Tumor laction	*n*	124	
	Right (%)	37 (29.8)	
	Left (%)	87 (70.2)	
Tumor size (cm)	*n*	123	
	Median (range)	4.2 (0.70-15.00)	
Histologic differentiation	*n*	121	
	Well/moderate (%)	108 (89.3)	
	Poor/undifferentiated (%)	13 (10.7)	
Tumor depth	*n*	128	
	T1+T2 (%)	33(25.8)	
	T3+T4 (%)	95(74.2)	
Dukes' stage	*n*	129	
	A+B+C (%)	103 (79.8)	
	D (%)	26 (20.2)	

### Fecal samples and colonic tumor specimens

Solid fecal samples were collected from the 129 patients prior to any surgical or chemical therapy, as described in our previous report [20]. Briefly, approximately 0.5 g of each fecal sample was preserved in 1 mL of guanidinium thiocyanate buffer (10 mM Tris, pH 7.4; 200 mM NaCl; 1 mM EDTA, pH 8.0; 4 M guanidinium thiocyanate; 1% β-mercaptoethanol) at -80°C until needed. In addition, fecal samples were collected from 85 healthy controls (age range, 32-91 years; mean age, 57 years; 42 males and 43 females) (Table 1) who were examined by colonoscopy and had no inflammatory bowel conditions. Colonic tumor specimens from six CRC patients (patients 01-06; age ≤ 60 years) were snap-frozen in liquid nitrogen immediately after surgery. The tissues were then directly homogenized for Western blot analysis, according to routine procedures.

### Quantitation of fecal CK19 expression

Fecal total RNA and cDNA were obtained as described in our previous reports [20,22]. Briefly, 1 μg of fecal total RNA was reverse transcribed using oligo(dT) primers (One-Step RT-PCR kit;Bioman, Taiwan, ROC), according to the manufacturer's instructions. The resulting cDNA sample was used to perform quantitative PCR (qPCR). Levels of fecal CK19 mRNA were measured using probes from the Universal Probe Library and TaqMan Master Mix in a LightCycler thermal cycler system, according to the manufacturer's instructions (Roche Diagnostics GmbH, Mannheim, Germany). The primers used to amplify CK19 (AF202321) were 5'-TTGTCCTGCAGATCGACAAC-3' (forward) and 5'-GCCTGTTCCGTCTCAAACTT-3' (reverse), which were used in combination with Universal Probe #71. The primers for 18s rRNA (X03205) were 5'-CTCAACACGGGAAACCTCAC-3' (forward) and 5'-CGCTCCACCAACTAAGAACG-3' (reverse), which were used in combination with Universal Probe #77. The LightCycler software (version 4.05, Roche Diagnostics) was used to analyze the qPCR kinetics and to calculate quantitative data. Each run included a diluted (512-fold) cDNA from HT-29 cells, which was used as a positive control to standardize the run-to-run differences in RNA quantity.

### Immunodetection of CK19 protein in colonic cell lines and tissues

Four different-staged CRC cell lines (SW480: ATCC number CCL-228; LS 174T: ATCC number CL-188; LoVo: ATCC number CCL-229; HT-29: ATCC number HTB-38) were cultured to harvest cellular protein. All cultured cells were maintained in Dulbecco's Modified Eagle's Medium with 5 mM glutamine, according to routine cell culture procedures. The cellular lysate of each cell line was harvested by scraping off the cells in radioimmunoprecipitation assay (RIPA) buffer (50 mM Tris-HCl (pH 7.4), 1 mM EDTA, 150 mM NaCl, 1% Nonidet P-40, 0.5% sodium deoxycholate, and proteinase inhibitors). Total protein concentration was determined using the Bradford protein assay kit (Bio-Rad Laboratories, Hercules, CA), according to the manufacturer's instructions. For each sample, 5 μg of protein was mixed with reducing NuPAGE SDS sample buffer (Life Technologies, Carlsbad, CA), denatured for 10 min at 95°C, separated by 15% SDS-PAGE, blotted onto a polyvinylidene difluoride membrane (Millipore, Billerica, MA), and probed with mouse anti-human CK19 (1:500; sc-6278; Santa Cruz Biotechnology, Santa Cruz, CA) or rabbit anti-human Actin (1:500; sc-1616-R; Santa Cruz Biotechnology), following standard procedures. Blots were then incubated with anti-mouse (for CK19) or anti-rabbit (for Actin) secondary antibodies (0.2 μg/mL) conjugated to horseradish peroxidase. All Western blots were developed using the Western Blot Chemiluminescence Reagent (PerkinElmer Life and Analytical Sciences, Waltham, MA), according to the manufacturer's instructions.

The two CRC cell lines LS 174T and LoVo were grown on glass coverslips in a 24-well plate and were fixed with 4% paraformaldehyde in PBS (1.37 mM NaCl, 2.7 mM KCl, 4.3 mM Na_2_HPO_4_, 1.4 mM KH_2_PO_4_, pH 8.3) and stained for cellular CK19 using fluorescent immunocytochemistry. Fixed cells were blocked in PBS with 1.5% normal horse serum (S-2000; Vector Laboratories, Burlingame, CA) for 30 min at room temperature and were then probed with mouse anti-human CK19 (1:100) antibody for 16 h at 4°C:. Unbound primary antibody was removed by two 5-min incubations with PBS, which were followed by incubation with Cy3-conjugated goat anti-mouse antibody (1:200; AP124C; Millipore) for 1 h at room temperature. Cells were washed with PBS and DNA was stained with 4',6' diamidino-2-phenylindole (DAPI). The stained samples were then dehydrated, mounted, and analyzed using a Nikon Eclipse 80i fluorescence microscope (Nikon Instruments, Melville, NY).

To evaluate the expression of CK19 in colonic tissues, Western blot analysis was performed using the protocol described for the cell line experiment, with some modifications. Briefly, frozen CRC tissues were pulverized in RIPA buffer using a tissue homogenizer and a sonicator. The tissue lysates were then separated by SDS-PAGE and immunoblotted as described above.

### Statistical analysis

Data were statistically analyzed using the SPSS software (SPSS, Chicago, IL). The Kruskal-Wallis test was applied to assess the presence of significant differences in fecal CK19 expression across the groups of healthy controls and patients without (M0 stage) and with (M1 stage) distant metastasis. Two groups were compared using the Mann-Whitney *U *test. A *post hoc *analysis was performed using Dunn's multiple comparison for the significant Kruskal-Wallis tests. The Pearson's correlation coefficient and a percent matching statistic were assessed to correlate the level of fecal CK19 expression with patient's clinicopathologic features, or with the metastatic rates of patients in different age intervals. Significance was set at *p *< 0.05.

## Results

### Age-related expression of fecal CK19 mRNA in patients with CRC

**Figure 1:**
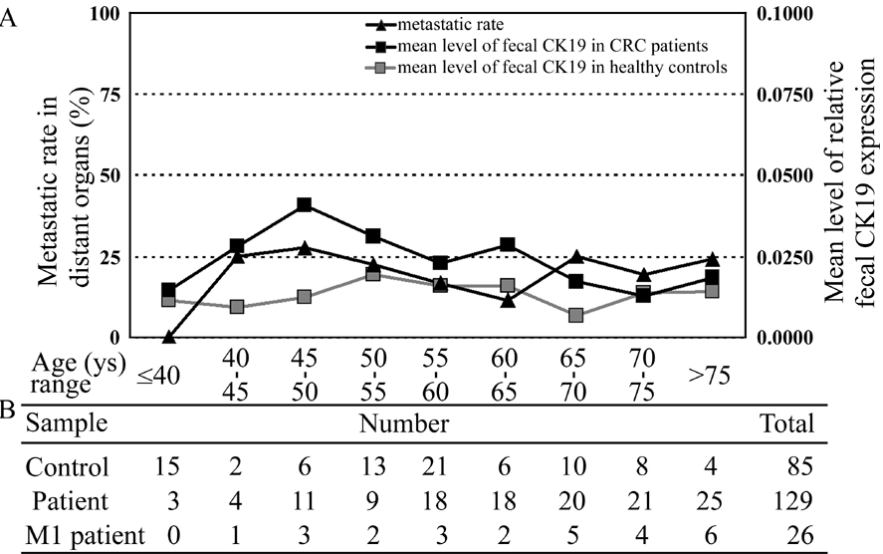
**Correlations between the mean level of fecal CK19 mRNA and the distant metastatic rate for CRC patients**. Each mean level of fecal CK19 mRNA was calculated from patients (black square) or healthy controls (gray square) in the same age interval as indicated. A metastatic rate (black triangle) was calculated by the following formula: number of patient with M1 stage/total number of patient in the same age interal ×100. Ages ranges of these subjects were 32--90 years for CRC patients and 32--91 years for healthy controls.

The median normalized level of fecal CK19 mRNA was 0.0073 (range, 0.0000-0.0665) in 85 healthy controls, 0.0146 (range, 0.0000-0.1528) in 129 CRC patients, 0.0142 (range, 0.0000-0.1183) in 103 patients without distant metastasis (M0 stage), and 0.0159 (range, 0.0000-0.1528) in 26 patients with distant metastasis (M1 stage). In analyses of clinicopathologic features, the fecal CK19 expression was netatively correlated with patient's age (*p *= 0.025), but positively correlated with tumor depth (*p *= 0.023). Other features including gender (*p *= 0.578), tumor location (*p *= 0.908), tumor size (*p *= 0.828), and differentiation (*p *= 0.581) were not significantly correlated with fecal CK19 expression. As shown in Figure 1, mean values of fecal CK19 mRNA from patients and from healthy controls, and the distant metastatic rate of patients were depicted according to the age interval. Among the 45 younger patients (age ≤ 60 years), patients aged between 45 and 50 years (45 ≤ age < 50) had the highest metastatic rate (27.3%, 3 of 11) in distant organs, whereas no metastasis were found in the youngest patient group (age ≤ 40 years). Additionally, the degree of consistency between the mean level of fecal CK19 mRNA and the distant metastatic rate was up to 89% (*p *= 0.042) in younger age groups (age ≤ 60 years) and was low in older age groups (age > 60 years) (-71%, *p *= 0.292). The mean value profiles of fecal CK19 mRNA from patients and healthy controls didn't show any consistency either in the younger or older age groups.

**Figure 2:**
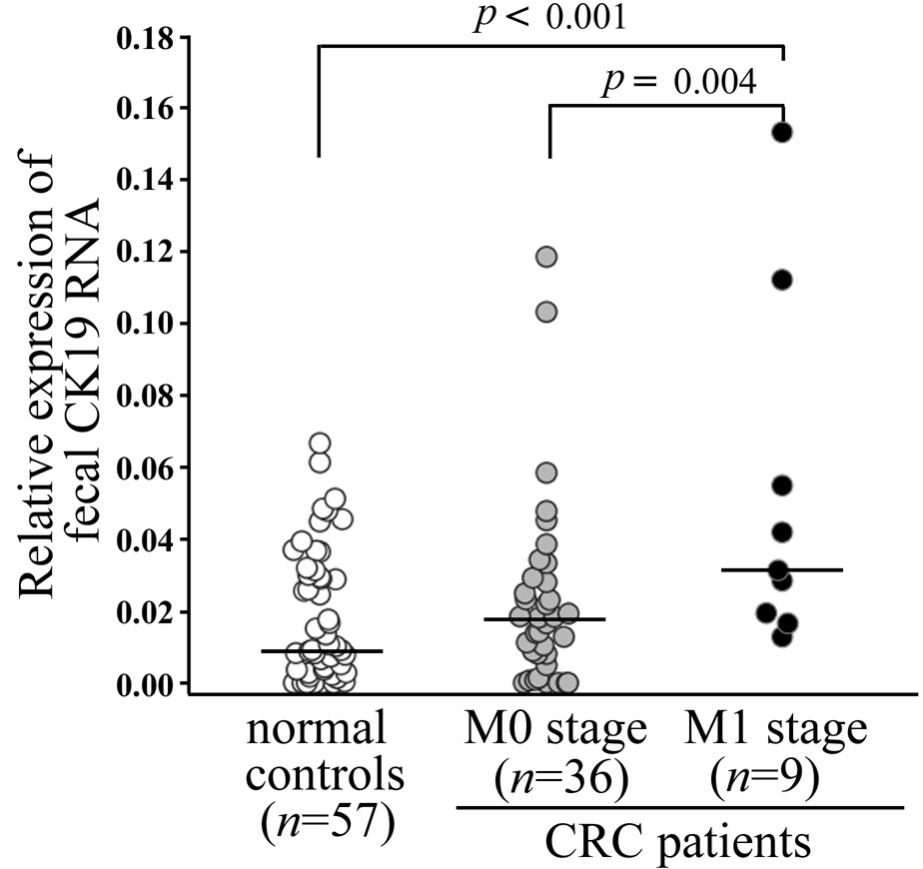
**Relative expression of fecal CK19 from younger patients and age-matched healthy controls**. The levels of fecal CK19 were quantified by quantitative real-time PCR from who were under the age of 60, including 57 healthy controls, 36 patients with M0 stage, and 9 patients with M1 stage. The relative expression of fecal CK19 mRNA was normalized by dividing it by that for 18s rRNA for each fecal sample. A significant difference (*p *< 0.05) determined by Dunn's multiple comparison was directly indicated as *p *value. The horizontal lines represent the median values.

As listed in Table 2, the younger patients with M1 stage (*n *= 9) had significantly higher fecal CK19 expression levels when compared with the older metastatic patients (*n *= 17), as assessed by the Mann-Whitney *U *test (*p *= 0.002). This significant difference of fecal CK19 mRNA correlated to the patients' ages was not found in the patients with M0 stage (*p *> 0.05). However, a significant difference was also seen across these younger groups by the Kruskal-Wallis test. (*p *= 0.004) The subsequent post hoc test in these younger subjects revealed that patients with M1 stage (*n *= 9) had significantly higher expression of fecal CK19 mRNA when compared with healthy controls (*n *= 57) (*p *< 0.001) or with patients with M0 stage (*n *= 36) (*P *= 0.004) (Figure 2). In contrast, no significantly different levels of fecal CK19 expression were noted from groups of older subjects (age > 60 years), such as patients with M0 (*n *= 67) and M1 stages, (*n *= 17) or healthy controls (*n *= 28) and patients with M1 stage.

**Table 2:** Median levels of fecal CK19 mRNA from different age groups of CRC patients

	Relative quantitation		
		
M stage	≤ 60 years	> 60 years	*p *value
M0			
*n*	36	67	
Median	0.0173	0.0135	> 0.05
Range	0-0.1183	0-0.1134	
M1			
*n*	9	17	
Median	0.0313	0.0117	0.002
Range	0.0128-0.1528	0-0.0994	

### Expression pattern of CK19 in various colonic cell lines

**Figure 3:**
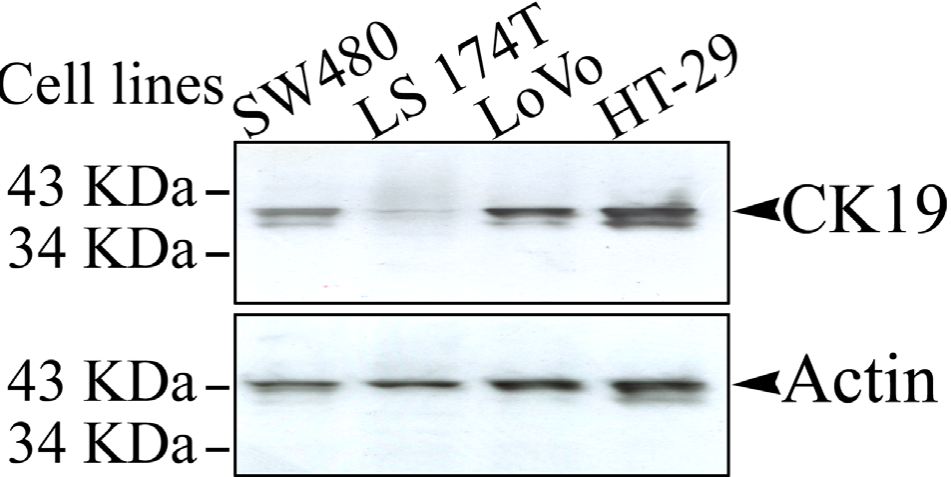
**Western blot analysis of endogenous CK19 expression in colonic cell lines**. All CRC cell lines presented with heterotypic bands at 40 KDa (CK19) and ~37 KDa. Bands from two early-staged cells (SW480 and LS 174T) were weaker than those from two late-staged cells (LoVo and HT-29). The molecular weight of Actin was 43 KDa. Antibodies, mouse anti-human CK19 antibody or rabbit anti-human Actin antibody.

**Figure 4:**
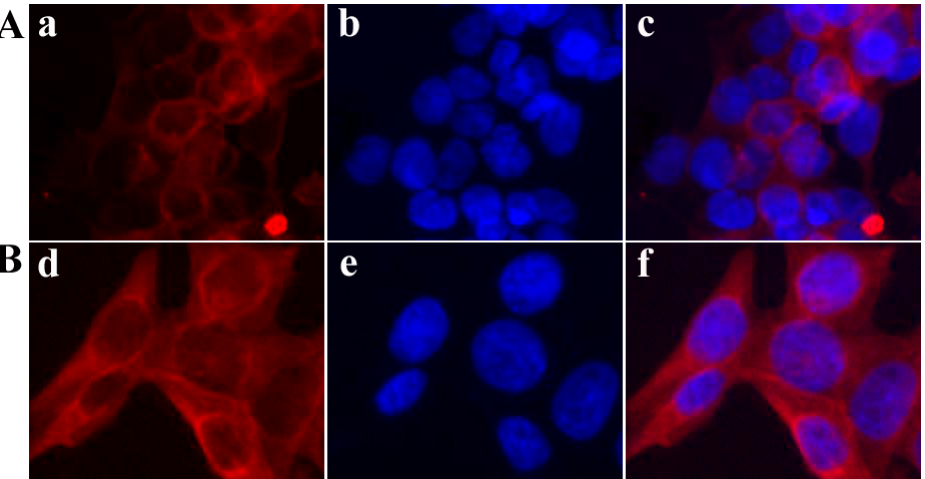
**Fluorescent immunocytochemistry of CK19 protein in CRC cell lines**. **A**: the early-staged cell line, LS 174T cells (200 ×). **B**: the late-staged cell line, LoVo cells (200 ×). Both the cells were stained for cellular CK19 (**a **and **d**) with mouse anti-human CK19 antibody. Secondary antibody, Cy3-conjugated goat anti-mouse antibody. **b **and **e**, nuclear stain (DAPI) of cells from **a **and **d**. **c**, merged image from **a **and **b**; **f**, merged image from **d **and **e**.

Based on the correlation observed between the levels of CK19 expression and the age of the patient, we chose four regular CRC cell lines (SW480, from a 50-year-old male; LS 174T, from a 58-year-old female; LoVo, from a 56-year-old male; HT-29, from a 44-year-old female) from donors who were all under the age of 60 and determined their endogenous levels of CK19 expression (40 KDa) using an immunoblot analysis (Figure 3). The two late-staged (LoVo and HT-29) CRC cell lines produced more CK19 protein when compared with the two early-staged (SW48 and LS 174T) cells, under similar levels of Actin protein. Moreover, one differentially expressed fragment (~37 KDa), which was smaller than the full-size CK19 protein, was detected in all CRC cell lines but with differential expressions. As shown in Figure 3, the expression level of this smaller fragment increased in accordance with the donor's clinical stage with the exception of that detected in SW480 cells. Immunofluorescent staining using a specific anti-human CK19 antibody showed a dramatic increase of CK19 protein in the cytosol of LoVo cells, which is in accordance with the immunoblot findings from the colon cell lines (Figure 4).

### Immunoblotting of CK19 protein in clinical colonic tissues

**Figure 5:**
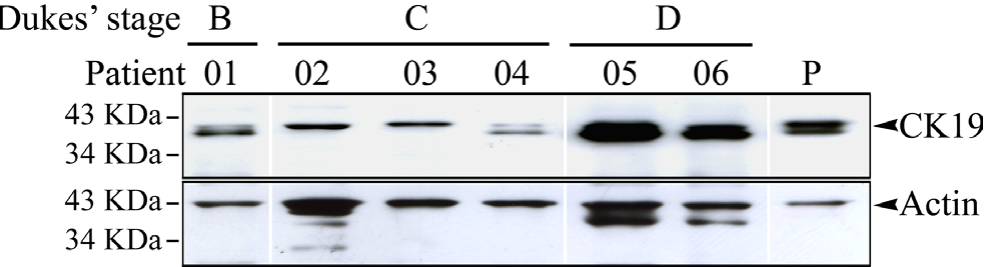
**Western blot analysis of endogenous CK19 expression in colonic tumor specimens**. Six colonic tumor specimens from one patient (01) at Dukes' stage B, three (02-04) at stage C, and two (05 and 06) at sage D were all under the age of 60 years. Two of patients (patients 02 and 03) presented with a monotypic band at 40 KDa (CK19) and others produced heterotypic bands at 40 KDa and ~37 KDa. The molecular weight of Actin was 43 KDa. Antibodies, mouse anti-human CK19 antibody or rabbit anti-human Actin antibody. P, HT-29 cells served as the positive control.

The same CK19 antibody was used to determine if clinical colonic tissues also exhibited altered levels of CK19 protein. As shown in Figure 5, colonic tumor specimens from six younger patients expressed CK19 in a pattern that was similar to that of the CRC cell lines (Figure 3) and was in accordance with their clinical stages. Even though the levels of CK19 protein (40 KDa) were not significantly different among these tumor tissues, the smaller fragment (~37 KDa) was upregulated in the two patients with Dukes' stage D (patient 05, a 52-year-old male; patient 06, a 49-year-old female).

## Discussion

Detection of CRC cells in feces using qRT-PCR may be developed for clinical diagnosis [18]. Because of the deregulation of apoptosis in CRC cells, cancer cells in feces are thought to be more stable than other cells sloughed from normal mucosa [23] and could even represent a target for the molecular diagnosis of CRC [24]; therefore, fecal molecules from exfoliated colonic cells are currently being developed as potential markers for colonic neoplasia [20].

We reported several fecal molecules that are differentially expressed in CRC patients [21,22], and demonstrated that CK19 expression in feces is possibly correlated with CRC metastasis [20]. Moreover, CK19 has been used extensively as a marker of micrometastasis and for the detection of circulating tumor cells in many cancers [25]. Recent studies showed that cancer patients with increased CK19 expression have lower survival rates [26]. In CRC patients, the levels of CK19 expression in the blood are associated with the presence of micrometastases [27,28].

In the present study, we determined that both tumor depth and distant metastasis of CRC patients were significantly correlated with higher fecal CK19 expression levels. The similar results were also reported by Wang et al. in blood [29]; however, our data are more specific in CRC because we using the samples directly from colonic tract [18]. In addition, this significant difference was only found in CRC patients aged less than 60 years of age. Among these younger patients, we also found that the levels of fecal CK19 mRNA varied according to the distant metastatic rate in each age interval. In particular, CRC patients aged between 45 and 50 years had both the highest mean value of fecal CK19 expression and distant metastatic rate, whereas the age-matched healthy controls had maximum fecal CK19 expression between 55 and 60 years of age. This means that the expression of fecal CK19 mRNA can be detected by qRT-PCR, and is significantly correlated with distant metastasis in CRC patients younger than 60 years. To our knowledge, very few reports have discussed the correlation between the expression levels of CK19 mRNA and the patient's age, especially in CRC. Li et al. reported that CK19 expressed in normal colon epithelial tissues is an aging-upregulated protein [30] and Kim et al. demonstrated a higher positive rate of fecal CK19 by immunodetection is found in gastrointestinal cancer and inflammation [31]. Recently, Pontiggi et al. suggested that cells expressing CK19 represent a subpopulation of basal keratinocytes in neonates and young children [32]. However, this is the first report that demonstrates that the expression levels of CK19 mRNA in feces of patients younger than 60 years may represent an underlying colorectal malignancy because of the prior exclusion of inflammatory bowel coditions. As reported by Karsten et al. and Dozois et al., who found that young people diagnosed with CRC are more likely to be in the later stage of the disease [17,33], we also found that the patients diagnosed with distant metastasis (9 of 45; 20%) were younger than 60 years. If we setting the cut-off value of 0.0142 (median value of patients with M0 stage), our data resulted in a high sensitivity 0.89 (95% CI, 0.79-0.99) but a low specificity 0.47 (95% CI, 0.36-0.58) for predicting young patients with M1 stage. This seems to correspond to the purpose of molecular screening and agree with Lemmon and Gardner who reported that a signature with high sensitivity but perhaps low specificity may be preferred in the clinical laboratory [34]. Because of the clinical significance of the association between fecal CK19 expression and the age of the patients, we used four CRC cell lines, and six colonic tumor tissues from young donors for immunodetection of the CK19 protein. Our immunoblot results revealed that the stage-related expression of CK19 was detected in certain young CRC cells. Moreover, the expression difference in the two early-staged cell lines, SW480 and LS 174T, was possibly caused by a recurrence of the malignancy and metastasis in the donor of SW480 cells later. These results suggest that increased awareness should be promoted in young patients who exhibit high expression levels of fecal CK19 mRNA, and that an aggressive CRC treatment should be implemented for these patients. This upregulation of CK19 was also observed LoVo cells when compared with LS 174T cells, using immunofluorescent imaging.

Additionally, a smaller fragment was present in the CRC cell lines and some colonic tissues, as assessed by immunoblot analysis. This result is in agreement with other studies, which describe the presence of a similar two-fragment pattern in immunoblots of CK19 [35]. We predicted that the size of the smaller fragment was approximately 37 KDa. We suggest that this is possibly the CYFRA21-1 fragment of CK19 because of the similar molecular weight reported by Satoh et al. [36]. This fragment could be evaluated in blood to detect non-small cell lung cancer [37], in saliva to detect oral squamous cell carcinoma [38], and in urine to detect bladder cancer [39]. Although we can not discriminate CYFRA 21-1 from CK19 via the qRT-PCR in feces, the fecal CK19 can still partially reflect the sloughed CRC cells because of a coincidence between the data of feces and colonic tumor specimens (data not shown). As reported for other types of cancer, the molecular assessment of CK19 expression levels using mRNA detection (qRT-PCR) was more sensitive, and was significantly associated with poor disease prognosis [40]. Our data are also consistent with the conclusions of Silva et al. and Denis et al., who demonstrated that the analysis of CK19 expression can be used to determine the presence of micrometastatic cells, even if these authors performed their assays in blood samples [27,28].

## Conclusion

The identification of occult metastases by molecular screening should improve the prediction of disease outcomes for CRC patients [5,28]. In conclusion, our results, together with those of others, indicate that high levels of fecal CK19 mRNA represent a potential marker for colorectal malignancy in younger patients (age ≤ 60 years), which implies that special awareness should be promoted among younger patients with high levels of fecal CK19 mRNA, and that aggressive treatment should be implemented for these patients. Additionally, the fecal CK19 molecule offers new insights into the study of the molecular alterations of CRC through fecal analysis.

## Abbreviations

CRC: colorectal cancer; CK19: cytokeratin 19; qRT-PCR: quantitative real-time reverse transcription polymerase chain reaction; CYFRA 21-1: CK19 protein fragment.

## Competing interests

The authors declare that they have no competing interests.

## Authors' contributions

CCCha and SHY contributed the idea and did basic conceptual work; CJH prepared the paper, did laboratory work, and added conceptual ideas; CCChi supervised manuscript preparation and added conceptual ideas; SHY and SHC did patient recruitment; SP, CLL, and CML analyzed the data with the assistance of YYW (immunodetection); HLS and CCH did statistical analysis; CLL and RNY offered clinical information of colorectal cancer. All authors read and approved the final manuscript.

## Pre-publication history

The pre-publication history for this paper can be accessed here:

http://www.biomedcentral.com/1471-2407/9/376/prepub
